# Using Nonlinear Vibroartrographic Parameters for Age-Related Changes Assessment in Knee Arthrokinematics

**DOI:** 10.3390/s22155549

**Published:** 2022-07-25

**Authors:** Krzysztof Kręcisz, Dawid Bączkowicz, Aleksandra Kawala-Sterniuk

**Affiliations:** 1 Faculty of Physical Education and Physiotherapy, Opole University of Technology, 45-758 Opole, Poland; 2 Faculty of Electrical Engineering, Automatic Control and Informatics, Opole University of Technology, 45-758 Opole, Poland; kawala84@gmail.com

**Keywords:** arthrokinematics, signal processing, vibroartrography, joint motion quality

## Abstract

Changes in articular surfaces can be associated with the aging process and as such may lead to quantitative and qualitative impairment of joint motion. This study is aiming to evaluate the age-related quality of the knee joint arthrokinematic motion using nonlinear parameters of the vibroarthrographic (VAG) signal. To analyse the age-related quality of the patellofemoral joint (PFJ), motion vibroarthrography was used. The data that were subject to analysis represent 220 participants divided into five age groups. The VAG signals were acquired during flexion/extension knee motion and described with the following nonlinear parameters: recurrence rate (RR) and multi-scale entropy (MSE). RR and MSE decrease almost in a linear way with age (main effects of group p<0.001; means (SD): RR=0.101(0.057)−0.020(0.017); and MSE=20.9(8.56)−13.6(6.24)). The RR post-hoc analysis showed that there were statistically significant differences (p<0.01) in all comparisons with the exception of the 5th–6th life decade. For MSE, statistically significant differences (p<0.01) occurred for: 3rd–7th, 4th–7th, 5th–7th and 6th life decades. Our results imply that degenerative age-related changes are associated with lower repeatability, greater heterogeneity in state space dynamics, and greater regularity in the time domain of VAG signal. In comparison with linear VAG measures, our results provide additional information about the nature of changes of the vibration dynamics of PFJ motion with age.

## 1. Introduction

Improved quality of life and rapid civilisation development have increased life expectancy [1,2,3]. In addition, extending life span together with decreasing births ratio result in population aging, in particular in highly developed countries [2,3,4]. Based on this, it is possible to predict that people at the age of 60 and older will make up 25% of the population [4,5]. Progress in medicine also contributes to extended life span in an aging society and requires more and more advanced and modern diagnostic methods [4,6,7,8,9]. Joints and their ligaments; elasticity loss; and capsular fibrosis are typical age-related physiological processes [2,10,11]. Appropriate medical diagnosis may lead to proper therapies; however, as of today, the standard diagnostic process is typically limited to X-rays, medical interview, and physical examination. These methods do not provide very precise information regarding the extent of potential degenerative or age-related changes in soft tissues, which could be estimated with magnetic resonance imaging (MRI); however, due to a high cost of this specific diagnostic method, it is seldom requested by medical professionals [2,12]. Unfortunately, the vast majority of currently used imaging methods, including those more sophisticated and expensive (including e.g., MRI) do not allow the assessment of the quality of joint motion [2].

One of the methods applied for joint condition assessment is the vibroarthrography (VAG), which works in such a way that the articular surfaces motion-generated vibroacoustic signals are recorded [2,13]. The VAG signals recorded from joints with some abnormalities have different waveform patterns in comparison to those obtained from the healthy ones, which may be a result of vibration magnitude increase in relation to articular cartilage degeneration or damage [2]. Disorders within the articular surfaces are associated with a deterioration of the quality of movement and manifested by the occurrence of crepitations, which allows the use of the vibroacoustic signal to assess the condition of the articular surfaces in the course of disorders of their functionality [2,13].

Imaging techniques, despite being expensive, provide information regarding the structure of analysed joints, but do not allow for assessing its functioning factors. These can be assessed during physical examination; however, this one is of a highly subjective nature [2,14,15,16]. As mentioned before—crepitations are regarded as a manifestation of degenerative changes or abnormalities in joints [2,17,18,19,20,21]. The use of VAG can be considered a more sensitive and accurate diagnostic method [2,17,20]. Biomechanical and morphological alterations are claimed to be related with a joint degenerative disease, such as inter alia: cartilage degeneration and decreased rheological properties of synovial fluid [16,22,23,24]. In addition, chondral deteriorations correspond to the level of vibroacoustic emission based on vibroarthrography (VAG) signals—analysis, where the affected knees produce acoustic emissions with a greater frequency, higher peaks, and longer duration compared to those obtained from the healthy ones [17,25,26,27].

The knee-joint is one of the most loaded joints in the human body and is prone to various injuries and potential early degeneration. The patellofemoral joint (PFJ) plays a very important role in the knee extensor mechanism. Its part—the patella—is known as the largest sesamoid bone, which acts as a shield for the anterior trochlea and protects it against excessive friction between the quadriceps tendon and the femoral condyles [28,29,30,31,32]. As far as joint motion assessment is concerned, one can distinguish some quantitative and qualitative methods [25,28,33,34,35,36,37]. The quantitative assessments include, among others, the use of the electro-goniometer or arthrometer, but these have some limitations, as the evaluation focuses mainly on palpation in order to check the motion smoothness with regard to the crepitus presence or absence. Such assessment is frequently imprecise [25,28,36,37,38,39].

Based on our previous clinical experience and on a thorough literature study—the vibroarthrography (VAG) can be successfully applied for evaluation of arthrokinematic motion quality [25,28,37], as the vibroacoustic emission level closely corresponds to the chondral deterioration degree [28]. In addition, the osteoarthritis (OA) affected knees produce vibroacoustic emissions with a greater frequency, higher peaks, and longer duration compared to the healthy ones [28,33,40,41,42]. In addition, the VAG signals can be helpful in the PFJ particular disorders differentiation, due to their specific disorder-related pattern character [25,28,33,37].

Despite the VAG method being still in development, it already shows very promising accuracy, sensitivity and specificity in joints affected with various disorders compared to the healthy ones [28,33,43,44]. As this method is relatively new, there are no strict requirements for using measuring equipment or for conducting examinations [28,45]. Moreover, the waveforms of VAG signals are characterised by non-stationarity and, in the above-mentioned studies, linear parameters were the basis for analysis. According to the principles of signal processing, nonlinear parameters are more appropriate descriptors for non-stationary time series. The nonlinear VAG parameters, such as recurrence rate (RR) and multi-scale entropy (MSE), allow evaluation of the quality of changes in the pattern of mechanical vibrations that appears with age; in particular, temporal structure of the signal characterized by heterogeneity and quasi-periodicity (RR) and the presence of repetitive patterns in a time series makes it more predictable (orderly) than a time series in which such patterns do not repeat (MSE).

We hypothesize that the use of nonlinear parameters will better reflect changes in the biomechanical environment of human synovial joints under ageing than linear parameters allow. Specifically, the recurrence rate (RR) and multi-scale entropy (MSE) will decrease in a linear manner with age. The aging processes should result in changes of VAG signal temporal structure characterized by greater heterogeneity in state space dynamics (RR) and higher repeatability (MSE) in the time domain.

## 2. Materials and Methods

In this study, the authors focused on nonlinear VAG parameters, such as recurrence rate (RR) and multi-scale entropy (MSE). Impaired quality of joint motion can be a clinical sign for articular surfaces’ disorders [2]. In this work, the vibroacoustic signal analysis was applied for articular surfaces’ assessment of knees. We reanalyzed the VAG data that were used in [46] and consisted of 220 study participants divided into five groups, depending on their age.

### 2.1. Study Participants

In our experiments, the study group consisted of the locomotor system dysfunction prevention program participants at the Institute of Physiotherapy, Opole University of Technology in 2012–2013. The group contained 220 healthy individuals—127 females and 93 males. The participants were divided into five age-related groups: ‘20–29’, ‘30–39’, ‘40–49’, ‘50–59’ and ‘60–69’, as presented in Table 1.

Study exclusion criteria were as follows, in order to prevent any disorder-related artifacts occurrence:

- any diagnosed knee disorders;
- post-traumatic syndromes;
- neurological disorders;
- functional limitations;
- pain feeling.

All study participants gave their informed consent and the testing was conducted in accordance with the Declaration of Helsinki, and approved by the Bioethics Committee of the Opole Medical Chamber in Opole, Poland, (No. 202 of 6 June 2013) for studies involving humans.

During experiments, assessment of the PFJ function for each knee was performed with the use of an acceleration sensor placed 1 cm above the patella apex and mounted with a double-sided adherent tape. The PFJ motion quality vibroarthrographic evaluation was based on tests lasting six seconds only. The procedure was the same as in [46]. Participants were tested in the sitting position, and each of the following tasks was repeated four times:

1. loose hanging legs with knees flexed at $90^{\circ }$;
2. full knee extension from $90^{\circ }$ to $0^{\circ }$;
3. knee reflexion from $0^{\circ }$ to $90^{\circ }$.

The constant velocities of both flexion and extension motions and measuring conditions were kept at the level of 82 beats per minute and measured with a metronome, while the knee joint angle was measured using an electrogoniometer placed on the knee lateral aspect with the rotation axis at the lateral femur condyle.

As the VAG signal might be affected (distorted) by the electrogoniometer placing due to its potential noise generation, this procedure was only used during experimental condition determination before the appropriate relevant tests took place.

**Figure 1:**
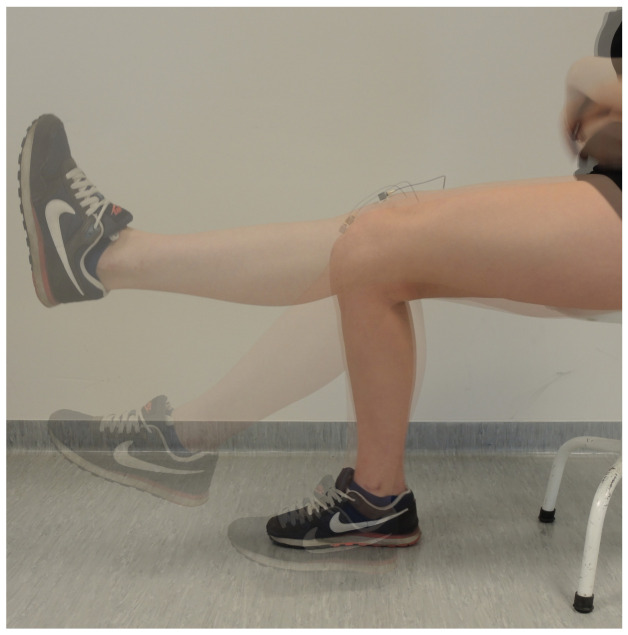
VAG signal’s recording.

For the purpose of VAG signals’ acquisition, a piezoelectric accelerometer (type 4513B-002, Bruel & Kjaer Sound Vibration Measurement A/S, Denmark) was applied and then the signal received by a transducer was passed on the low-noise measuring amplified input (Nexus by Bruel & Kjaer). The signals were recorded in the periodicity between 0.7 and 1000 Hz with the sampling frequency $F_{S}=10$ kHz and then filtered with a typical 4-order zero-phase Butterworth band-pass filter with the cut-off frequencies at 50 Hz and 1000 Hz. In Figure 1, a sample VAG recording is presented.

The nonlinear analysis of the VAG signals was based on the use of a quantitative representation of the graph’s recurrence rate and multi-scale entropy calculation. The RR repetition plots show a square matrix for which the elements correspond to times in which a certain state of the system repeats in the phase space (row coordinates and columns correspond to a specific pair of times or signal samples). The RR plot is a set of points, which represents events when the trajectory of a dynamical system passes through roughly the area itself in space (tolerance) [47].

The quantitative RR plot analysis is based on the calculation of a series of different parameters (RQA) from which we chose the RR parameter, which represents the percentage of repetitive system states normalized by the total number of states. RR allows evaluation of the quality of changes in the pattern of mechanical vibrations that appear with age, in particular signal temporal structure characterized by heterogeneity and quasi-periodicity. The RQA parameter was computed using the PyRQA Python package. Parameters of values for the RQA were chosen as follows: m = 6, time delay = 1, radius = 10%, Theiler corrector = 1, and distance nom = Euclidean.

Multiscale sample entropy analysis (MSE) is based on the calculation of the sample entropy of multiple signal time scales and determines its repeatability (regularity, complexity feature) over time. Presence of repetitive patterns in a time series makes it more predictable (orderly) than a time series in which such patterns do not repeat. When interpreting, however, the sensitivity of the MSE to outliers that may dominate the signal and cause IES not to indicate regularity, but, rather, the scale of occurrence of these values in the signal should have been taken into account. A detailed description of the MSE calculation can be found in [48].

In this paper, the MSE was calculated using the standard parameters: m = 2 and r = 0.15% SD in 30 time scales.

### 2.2. Statistical Analysis

Due to skewed distribution, a logarithm transformation was applied to recurrence rate (RR) parameters. Evaluation of all dependent variables (RR and MSE) was subjected to the five above-mentioned age groups (‘20–29’, ‘30–39’; ‘40–49’, ‘50–59’, ‘60–69’) × gender (Female and Male) × 2 sides (Left and Right leg) analysis of covariance (ANCOVA). Both body mass and height were included as covariates.

## 3. Results

When significant interactions were identified, Tukey analyses were applied as post-hoc tests. *p*-values ≤0.05 were considered as statistically significant. In Table 2, the obtained results of statistical analysis were presented.

**Figure 2:**
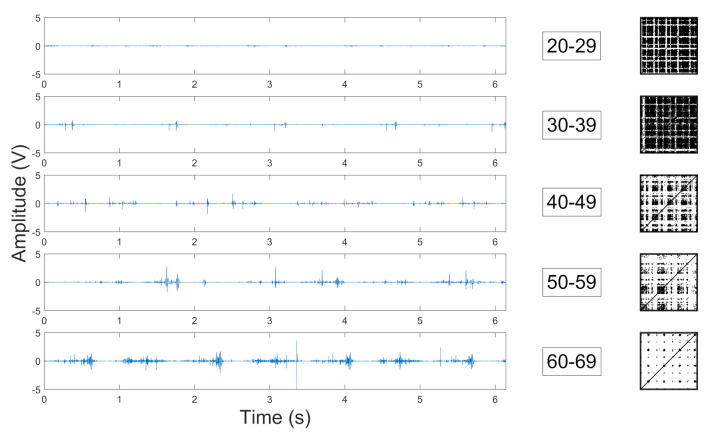
VAG signals’ representative sample wave-forms and their recurrence.

In Figure 2, representative sample wave-forms of vibroartrographic (VAG) signals collected during experiments and their recurrence plots were presented. The degree of repeatability is proportional to the number of black dots in the RR plots The post-hoc comparison is shown using connecting lines comparison of individual groups with the corresponding *p*-value on the horizontal axis.

**Figure 3:**
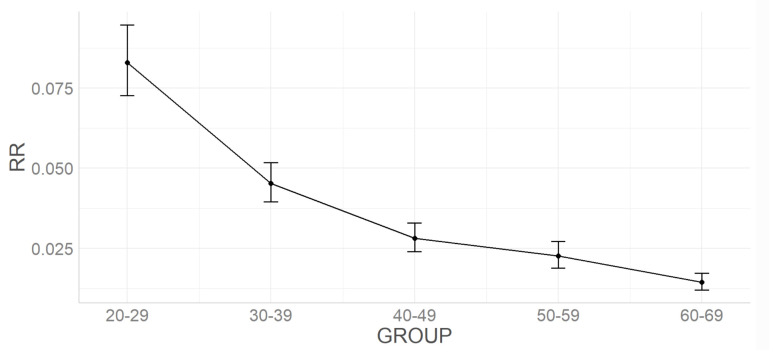
Mean and confidence intervals of VAG signal parameters in the further decades of life—for RR.

The RR approximately linearly decreases with age (main effect of GROUP F(4,418)=67.99,p<0.001). The post-hoc analysis showed that there were statistically significant differences (p<0.01) in all comparisons except the 5th–6th decade. In Table 3, the ANCOVA results were presented and illustrated with Figure 3.

The smaller the values of the sample entropy, the greater the regularity of the signal or the greater the number of extreme values. Statistical analysis was performed on the sum values across all temporal scales.

Table 2 contains the following values:

- Sum Sq—the sum of squares due to source;
- Mean Sq—the mean sum squares due to source;
- NumDF—numerator degrees of freedom;
- DenDF—denominator degrees of freedom;
- F value—the ratio of explained variance to unexplained variance;
- *p*—the probability of obtaining test results at least as extreme as the result actually observed, under the assumption that the null hypothesis is correct.

**Figure 4:**
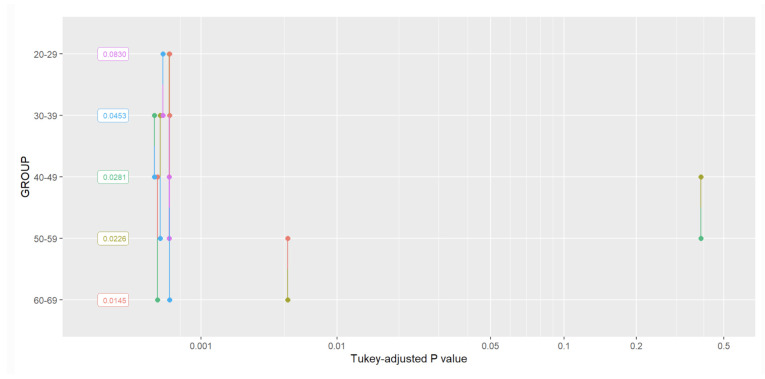
The RR Tukey analysis results.

In Figure 4, Tukey analysis results for the recurrence rate were presented.

**Figure 5:**
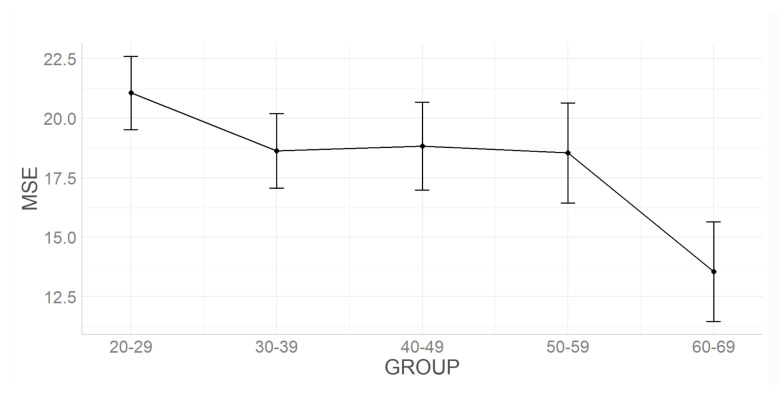
Mean and confidence intervals of VAG signal parameters in the further decades of life—for MSE.

The MSE also decreased with age (main effect of Group F(4,418)=8.34,p<0.001). The post-hoc analysis revealed that statistically significant differences (p<0.01) occurred for: 3rd–7th, 4th–7th, 5th–7th, and 6th life decades. In Table 4, the ANCOVA results for the MSE were presented and illustrated with Figure 5.

**Figure 6:**
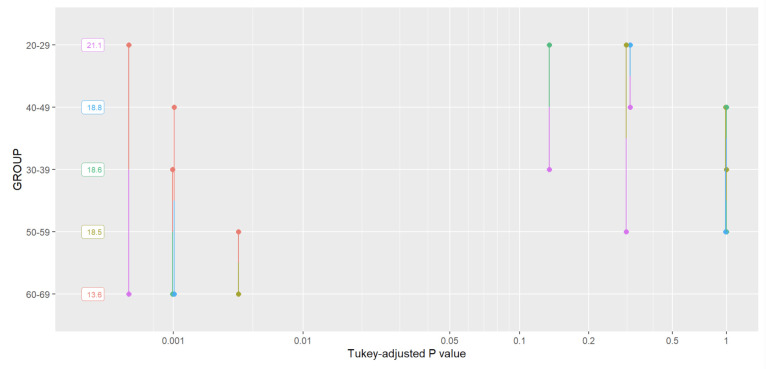
The MSE Tukey analysis results.

In Figure 6, Tukey analysis results for the multi-scale entropy were presented.

**Figure 7:**
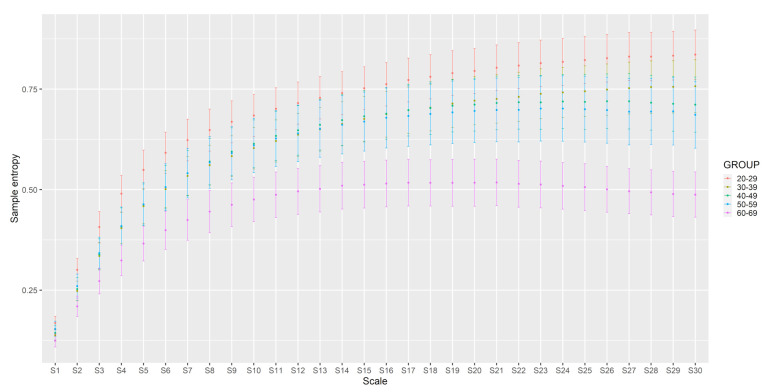
MSE analysis results in particular age groups.

Figure 7 illustrates the summary graph of the MSE analysis. The dots on the chart represent the averages, and the whiskers’ (thick lines) mean confidence intervals.

## 4. Discussion and Conclusions

The purpose of this study was to evaluate the age-related changes of quality of the knee joint arthrokinematic motion using nonlinear parameters of the vibroarthrographic (VAG) signal. Our assumption was that these parameters may be favorable descriptors of changes in the waveform of VAG, due to the non-stationary nature of signals. The results showed that RR and MSE decreased almost linearly with age. Whilst decreasing RR with age may suggest lower repeatability and greater heterogeneity in state space dynamics, MSE could indicate an increase in the regularity and repeatability of the signal in the time domain, but another interpretation is also possible: the MSE parameter is sensitive to signal outliers that cause a decrease of its value. This may indicate the presence of more sudden large amplitude vibrations in the signal.

The novelty of our work relies on the use of nonlinear parameters as descriptors characterizing VAG signals in particular decades of life. It is noteworthy that this is the first study evaluating aging-related changes in knee joint arthrokinematics using such analyses. The justification for the use of nonlinear parameters is largely due to the nature of the VAG signal, which is characterised by a non-stationary waveform. It is possible that filtering of the signal applied so far and described in the literature gives it some features of stationarity; however, it seems that, from the point of view of methodological correctness, the application of nonlinear parameters is more appropriate. Although their application does not result in a significantly higher level of discrimination of the tested groups, it seems that it can better reflect the changes occurring in the biomechanical environment of joints under the influence of ageing. In the previous study, it was difficult to explain the fact that, in subsequent decades of life, the values of linear parameters (both amplitude and frequency) show an increasing or decreasing tendency [46]. It is difficult to unequivocally explain which biological features in particular decades of life may be related to the successive wear of cartilage, and MSE may correspond to the viscoelastic properties of synovial fluid, the quality of which decreases significantly above the age of 60.

Based on previous research, we assume that the above-mentioned changes in vibroarthrographic signals are associated with several biomechanical impairments, associated with ageing and mechanical wear of joint structures [17,46,49]. All this can result in cartilage surface irregularity, fibrillation and softening. Furthermore, the ageing process may result in limited production (and degraded quality) of synovial fluid and hinder its diffusion in the joint cavity, negatively affecting lubrication of the articular surfaces [17,46]. Consequently, an increase in the coefficient of friction occurs during arthrokinematic motion, observed as increased vibrations registered in the VAG signal [23,46]. Of course, these are only hypotheses, and the explanation of these phenomena requires the design of new studies, taking into account studies in tissue morphology and biotribology.

In conclusion, the use of nonlinear parameters to evaluate the quality of the knee joint arthrokinematic motion extends knowledge of the nature of age-changes occurring in patellofemoral joints. It can be clearly seen that nonlinear parameters decrease almost linearly in particular decades of life, which may be related to the successive wear of cartilage and degradation of synovial fluid.

### Limitations of the Study

In our research, the electrogoniometer was used to control the proper range of motion (90−0−90) during patient training, but it was not used during the relevant measurements (recording of the VAG signal). The use of an electrogoniometer or additional accelerometric sensors causes the appearance of significant artifacts and noise in the recorded VAG signal, due to the extremely high sensitivity of the measurement sensors used.

Thus, the results presented here do not allow more in-depth analysis of the VAG signal with regard to the phases of motion (flexion-extension). Due to the different biomechanics of the joint during concentric and eccentric activity, it seems worthwhile to include measurement of the range of motion in future studies, for example, using optoelectronic systems.

## Figures and Tables

**Table 1:** Study participants’ anthropometric characteristics.

	Age (Years)				
Parameters	(20–29)	(30–39)	(40–49)	(50–59)	(60–69)
No. of subjects (M/F)	60(26/34)	56(24/32)	40(17/23)	31(13/18)	33(13/20)
Age (years) ^1^	23.8±2.4	34.9±3.0	45.5±2.7	55.0±3.0	64.8±2.9
Body-mass (kg) ^1^	69.3±11.8	71.5±14.0	73.5±12.1	74.6±11.5	72.9±9.0
Height (cm) ^1^	170.4±7.5	169.3±8.5	168.5±8.9	167.0±6.7	164.2±8.2
BMI ^1^	23.8±3.1	24.9±4.5	25.9±3.7	26.7±3.4	27.1±3.4

**Table 2:** Descriptive statistics of VAG parameters across age groups.

	20–29	30–39	40–49	50–59	60–69
(N = 120)	(N = 112)	(N = 80)	(N = 62)	(N = 66)	
RR					
Mean (SD)	0.101 (0.0565)	0.0591 (0.0450)	0.0378 (0.0358)	0.0296 (0.0243)	0.0198 (0.0176)
Median [Min, Max]	0.0919 [0.0126, 0.250]	0.0455 [0.00688, 0.236]	0.0287 [0.00373, 0.261]	0.0208 [0.00378, 0.119]	0.0146 [0.00247, 0.0953]
MSE					
Mean (SD)	20.9 (8.56)	18.6 (8.46)	18.4 (7.92)	18.1 (8.00)	13.6 (6.24)
Median [Min, Max]	20.9 [2.65, 41.8]	18.0 [3.25, 39.6]	18.6 [5.02, 46.2]	18.9 [1.92, 39.5]	13.4 [1.79, 30.4]

**Table 3:** Type III analysis of variance table with Satterthwaite’s method for RR.

	Sum Sq	Mean Sq	NumDF	DenDF	F Value	*p*
Group	137.939	34.485	4	418	67.9885	<2.2 × 10${}^{-16}$
Mass	0.047	0.047	1	418	0.0930	0.760490
Height	0.401	0.401	1	418	0.7899	0.374638
Side	0.090	0.090	1	418	0.1773	0.673923
Gender	0.919	0.919	1	418	1.8111	0.179100
Group:Side	2.103	0.526	4	418	1.0365	0.387976
Group:Gender	2.176	0.544	4	418	1.0724	0.369718
Side:Gender	3.491	3.491	1	418	6.8832	0.009019
Group:Side:Gender	2.942	0.736	4	418	1.4502	0.216631

**Table 4:** Type III analysis of variance table with Satterthwaite’s method for MSE.

	Sum Sq	Mean Sq	NumDF	DenDF	F Value	*p*
Group	1987.74	496.93	4	392.26	8.3372	1.836 × 10${}^{-6}$
Mass	108.64	108.64	1	384.75	1.8226	0.177793
Height	110.12	110.12	1	406.34	1.8475	0.174826
Side	46.14	46.14	1	363.63	0.7742	0.379507
Gender	419.81	419.81	1	416.08	7.0433	0.008261
Group:Side	56.74	14.19	4	365.63	0.2380	0.916785
Group:Gender	562.59	140.65	4	410.82	2.3597	0.052822
Side:Gender	18.74	18.74	1	365.04	0.3145	0.575292
Group:Side:Gender	144.13	36.03	4	361.84	0.6045	0.659617

## Data Availability

Samples of the compounds are available from the authors upon written request.

## Abbreviations

The following abbreviations are used in this manuscript:

PFJ	patellofemoral joint
VAG	vibroarthrography
RR	recurrence rate
MSE	multi-scale entropy
MRI	magnetic resonance imaging
